# Diagnostic Efficiency of Thyroglobulin in Lymph Node Fine-needle Aspiration Washout: A Systematic Review and Meta-analysis

**DOI:** 10.1210/clinem/dgaf467

**Published:** 2025-08-19

**Authors:** Chen-Yeh Yu, Edward Hung-Lun Chu, Che-Hsuan Lin, Yen-Chun Chen

**Affiliations:** Department of Otolaryngology, Taipei Medical University Hospital, Taipei 11031, Taiwan; Department of Medical Education, Taipei Medical University Hospital, Taipei 11031, Taiwan; Department of Otolaryngology, Taipei Medical University Hospital, Taipei 11031, Taiwan; Department of Medical Education, Taipei Medical University Hospital, Taipei 11031, Taiwan; Department of Otolaryngology, Taipei Medical University Hospital, Taipei 11031, Taiwan; Department of Otolaryngology, School of Medicine, College of Medicine, Taipei Medical University, Taipei 11031, Taiwan; Department of Otolaryngology, Taipei Medical University Hospital, Taipei 11031, Taiwan; Department of Otolaryngology, School of Medicine, College of Medicine, Taipei Medical University, Taipei 11031, Taiwan; Graduate Institute of Medical Sciences, College of Medicine, Taipei Medical University, Taipei 11031, Taiwan

**Keywords:** papillary thyroid cancer, fine-needle aspiration, thyroglobulin, thyroglobulin antibodies, thyroidectomy, metastatic lymph node

## Abstract

**Context:**

Cervical lymph node metastases are common in papillary thyroid carcinoma (PTC), causing recurrence and poor regional control, highlighting the need for accurate diagnostics. Although fine-needle aspiration with thyroglobulin washout (FNA-Tg) shows promise, its diagnostic performance and association with serum biomarkers across settings remain unclear.

**Objective:**

To assess the diagnostic performance of FNA-Tg for cervical lymph node metastases in PTC and its correlation with serum thyroglobin (s-Tg) and s-Tg antibody (s-Tg-Ab) levels.

**Data Sources:**

PubMed, Embase, Web of Science, Scopus, Cochrane Library, and Ovid Medline were searched for relevant studies.

**Study Selection:**

Studies enrolling PTC patients with cervical lymphadenopathy who underwent FNA-Tg pre- or postthyroidectomy were included. Studies involving non-PTC populations or lacking sufficient data for 2 × 2 diagnostic table construction were excluded.

**Data Extraction:**

Data were independently extracted by 3 researchers, and study quality was assessed by the Quality Assessment of Diagnostic Accuracy Studies-2 tool.

**Data Synthesis:**

FNA-Tg showed pooled sensitivity of 0.94 [95% confidence interval (CI), 0.91-0.96], specificity of 0.92 (95% CI: 0.88-0.94), and diagnostic odds ratio (DOR) of 174.20 (95% CI: 87.05-348.61), with an area under curve (AUC) of 0.98 (95% CI: 0.96-0.99). Postthyroidectomy, sensitivity increased to 0.96 (95% CI: 0.94-0.98), specificity to 0.93 (95% CI: 0.86-0.96), and DOR to 305.87 (95% CI: 127.99-730.93), with an AUC of 0.98 (95% CI: 0.97-0.99). In the group without thyroid gland present, sensitivity was 0.96 (95% CI: 0.93-0.97), specificity 0.93 (95% CI: 0.87-0.97), and DOR 300.27 (95% CI: 118.47-761.05), with an AUC of 0.98 (95% CI: 0.97-0.99). Likelihood ratio scattergrams and Fagan plots supported its discriminatory ability. FNA-Tg correlated weakly with s-Tg but not with s-Tg-Ab.

**Conclusion:**

FNA-Tg showed high diagnostic accuracy, especially after thyroidectomy, with minimal s-Tg-Ab interference, supporting its role in PTC surveillance.

Thyroid cancer is the most prevalent endocrine malignancy, and its incidence is increasing worldwide (1). Papillary thyroid cancer (PTC) represents approximately 80% of all cases of thyroid cancer (2). According to a systematic review conducted in 2023, the 5-year survival rate among patients with PTC is 95.3% (3). Patients with PTC frequently experience lymph node metastasis, which compromises locoregional control (4) and increases the risk of mortality (5). Accordingly, reliable diagnostic techniques are essential for the early detection of metastatic lymph nodes. PTC typically metastasizes to cervical lymph nodes. Approximately 30% to 80% of patients with PTC have lymph node involvement (6). Up to 20% of patients with PTC experience recurrence after treatment (7-9). Posttreatment surveillance is essential to prevent disease progression. American Thyroid Association guidelines recommend routine surveillance for patients who have undergone thyroidectomy. Routine surveillance for these patients should involve measurement of serum thyroglobulin (s-Tg) and serum thyroglobulin antibodies (s-Tg-Ab) every 6 to 12 months and neck ultrasound examinations every 12 months. Patients with elevated s-Tg concentrations should undergo whole-body radioiodine scanning, which is used to detect potential recurrence. In cases of suspected lymph node metastasis, fine-needle aspiration with thyroglobulin washout (FNA-Tg) is recommended (10).

Ultrasonography is the primary diagnostic modality for the detection, characterization, and evaluation of thyroid nodules and for the detection of cervical lymph node metastasis. Ultrasound-guided fine-needle aspiration cytology is employed to assess the cytological characteristics and tumor status of cervical lymph nodes. Fine-needle aspiration cytology is reliable and has sensitivity rates of 85.6% and 60% to 80% and specificity rates of 71.4% and 70% to 90% for detecting tumors in thyroid nodules and lymph nodes, respectively (11). Its accuracy depends on factors such as the size and location of the lymph node and the experience of the operator (12, 13). The method produces false-negative results in 6% to 8% of cases and inconclusive results in 20% to 28.5% of cases (14). FNA-Tg can be used as a complementary diagnostic tool (15, 16).

Several review articles have discussed the reliability of FNA-Tg as an adjunctive tool for confirming cervical lymph node metastasis in patients with PTC (14, 17). Nevertheless, a systematic analysis of the tool's diagnostic validity and accuracy has not been performed (14). Additional research is warranted whenever advancements in testing techniques are developed. Accordingly, this study systematically reviewed the literature to determine the diagnostic validity and accuracy of FNA-Tg for confirming cervical lymph node metastasis in patients with PTC undergoing cervical lymphadenopathy. The diagnostic validity and accuracy of FNA-Tg in patients with PTC and concurrent lymphadenopathy before or after thyroidectomy were compared. Additionally, this study examined whether FNA-Tg measurements are correlated with s-Tg and s-Tg-Ab concentrations.

## Methods

### Study Design

#### PICO strategy

We used the PICO strategy:

P (patient): patients with PTC and concurrent cervical lymphadenopathy before or after thyroid surgery (total thyroidectomy, hemithyroidectomy, or partial thyroidectomy). No restrictions on country, sex, or ethnicity were placed in the inclusion of studies.
I (intervention): the presence of thyroglobulin in metastatic lymph nodes affects the diagnostic accuracy of PTC by fine-needle aspiration.
C (compare): Not presence of thyroglobulin in metastatic lymph nodes by fine-needle aspiration but s-TG, s-Tg-Ab, and antithyroglobulin antibodies can be used for diagnosis of PTC.
O (outcomes): the diagnostic efficacy and accuracy of FNA-Tg for recurrent or metastatic PTC in terms of sensitivity, specificity, positive diagnostic likelihood ratio (DLR+), negative diagnostic likelihood ratio (DLR−), diagnostic odds ratio (DOR), positive predictive value (PPV), negative predictive value (NPV), hierarchical summary receiver operating characteristic (HSROC), and area under the curve (AUC).

In accordance with the PRISMA 2020 statement, we conducted a systematic literature search using PubMed, Embase, Web of Science, Scopus, Cochrane Library, and Ovid Medline databases, with data retrieved up to November 2024. We included articles involving patients with PTC and concurrent cervical lymphadenopathy who had undergone cervical lymph node FNA-Tg due to the suspicion of metastatic PTC and whose final diagnosis had been confirmed using surgical pathology. We excluded studies involving patients with types of thyroid cancer other than PTC, such as follicular or other poorly differentiated thyroid cancer, and studies that lacked data enabling us to construct a 2 × 2 table detailing the rates of false positives, false negatives, true positives, and true negatives.

### Study Selection

We applied Boolean logic in our search and used the following search terms: (thyroid cancer AND papillary) AND (fine-needle aspiration OR fine needle OR biopsy) OR (thyroglobulin OR antithyroglobulin) OR (lymphatic metastasis OR lymphadenopathy OR cervical lymph nodes) OR (thyroidectomy) OR (total thyroidectomy). When searching databases that did not allow for Boolean searching, we configured the built-in advanced search function to include the aforementioned search terms. The search employed the following keywords in each database to identify relevant studies: (papillary thyroid) AND (lymph node or nodal or cervical) AND (fine needle) AND (thyroglobulin). Original articles published in English between January 2008 and November 2024 were screened for eligibility. Three authors (C.-Y.Y., E.H.-L.C., and Y.-C.C.) independently conducted the literature search and evaluated the retrieved journal articles. Retrieved articles were pooled into an EndNote file (EndNote version 20, Clarivate, Philadelphia, PA, USA), and duplicates were removed by executing the “Find Duplicates” function in EndNote. The 3 authors independently screened remaining articles by title and abstract to exclude those that were irrelevant to the topic or did not satisfy the inclusion criteria. The full text of each of the remaining articles was then read, and articles that did not provide detailed and complete data were excluded.

### Data Extraction and Quality Assessment

All studies underwent a thorough review. The following data were extracted from each study by 3 independent researchers: name of first author; publication year; study design; country; number of patients; number of lymph node samples; sex ratio; age range; diagnostic criteria; targeted population; disease stage; initial or recurrent metastasis; type and volume of fine-needle aspiration washout solution; FNA-Tg cutoff value; presence of thyroglobulin antibodies in fine-needle aspiration washout fluid; presence of s-Tg; presence of s-Tg-Ab; and absolute numbers of true-positive, false-positive, false-negative, and true-negative test results; and PPV and NPV. The quality of the included studies was assessed using the Quality Assessment of Diagnostic Accuracy Studies-2 (QUADAS-2) tool (18). Methodological quality is assessed in the QUADAS-2 in terms of the patient selection, index test, reference standard, and flow and timing in a given study. Studies were classified as having low, high, or unclear risk of bias for each domain. The Grading of Recommendations Assessment, Development and Evaluation (19) approach was incorporated to evaluate the certainty of evidence and enhance the comprehensiveness of the analysis [Supplemental Table S1 (20), (Zenodo)].

### Statistical Analysis

Data were analyzed using STATA 18. A bivariate random-effects regression model was used to calculate the pooled sensitivity, specificity, DLR+, DLR−, DOR, PPV, NPV, and 95% confidence intervals (CIs). HSROC curves with pooled sensitivity and specificity findings were drawn. Heterogeneity was assessed using a Cochran Q and inconsistency (I^2^) index, with I^2^ values greater than 50% indicating significant heterogeneity. Bivariate boxplots were used to assess the presence of the threshold effect. Fagan plots with likelihood ratios were used to predict posttest probabilities. Publication bias was assessed using a Deeks’ funnel plot. *P* < .05 indicated statistical significance.

#### Subgroup analysis

To investigate potential sources of heterogeneity and enhance the clinical applicability of the findings, predefined subgroup analyses were conducted based on patient characteristics known to influence FNA-Tg performance. Prior studies have shown that FNA-Tg levels may be affected by s-Tg, s-Tg-Ab, and the presence or absence of remnant thyroid tissue. Accordingly, 4 clinically relevant subgroups were analyzed. The preoperative group consisted of patients who had not undergone thyroid surgery (n = 12 studies). The postoperative group comprised patients who had undergone total, hemi-, or partial thyroidectomy (combined groups 2.1, 2.2, and 2.3; n = 17 studies). The group with thyroid present [thyroid (+)] group consisted of patients who had remaining thyroid tissue (groups 1, 2.1, 2.2, and 2.4; n = 16 studies). The group in which thyroid was absent [thyroid (−)] comprised patients without thyroid tissue (group 2.3; n = 14 studies). These subgroup definitions were used to examine whether surgical status or thyroid tissue presence influenced the diagnostic accuracy of FNA-Tg and to assess the consistency of test performance across clinically distinct scenarios.

## Results

### Included Studies

**Figure 1.**
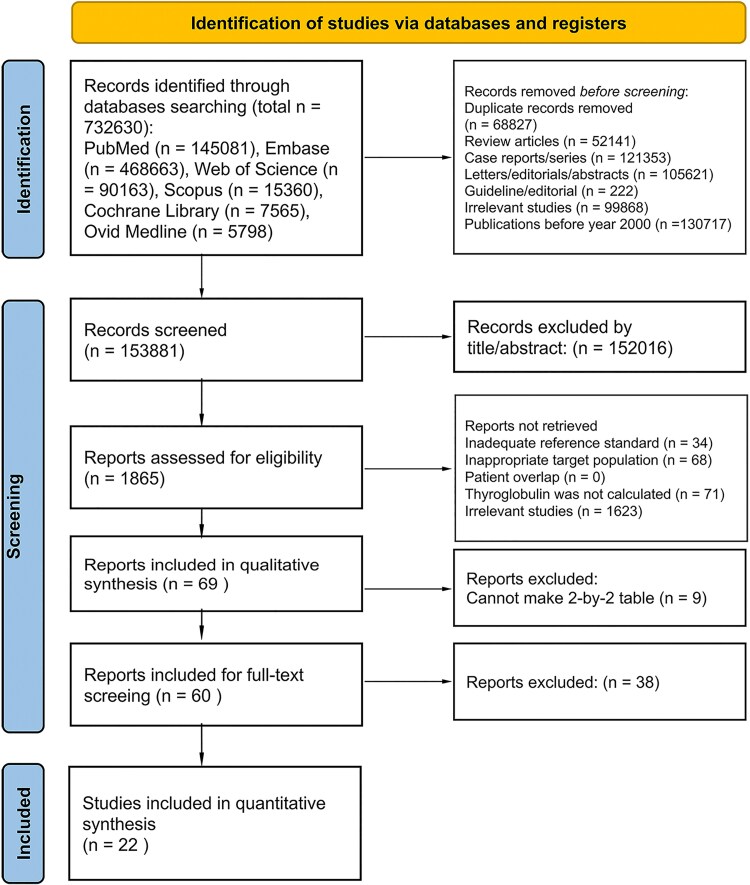
Study selection process.

A total of 732 630 records were initially retrieved from PubMed (145 081), Embase (468 663), Web of Science (90 163), Scopus (15 360), Cochrane Library (7565), and Ovid Medline (5798). After duplicates, reviews, case reports, letters, editorials, abstracts, guidelines, and unrelated studies were removed, 153 881 titles and abstracts remained for screening. In total, 152 016 of these titles and abstracts were deemed irrelevant. The remaining 1865 full-text articles were assessed for eligibility, and 1796 were excluded due to inappropriate reference standards, nontarget populations, patient overlap, missing data, or irrelevance. Of the 69 studies eligible for qualitative synthesis, 9 lacked sufficient data for 2 × 2 contingency tables to be constructed and were thus excluded. Following full-text screening, an additional 38 studies were excluded. Ultimately, 22 studies were included in the meta-analysis. The study selection process is illustrated in Fig. 1 (PRISMA 2020) (21). Study characteristics and qualitative data are summarized in Tables 1 and 2, respectively. Studies excluded in the full-text screening stage are listed in Supplemental Table S2 (20). (Zenodo)

**Table 1.** Summary of included study characteristics. Table summarizing characteristics of the included studies evaluating FNA-Tg in lymph node metastasis diagnosis. Key information includes study design, region, sample size, gender ratio, age, diagnostic criteria, surgical status, and disease recurrence status.

First author (year)	Study design	Region	Sample size	Sex ratio (female:male)	Age range/mean (years)	Diagnostic criteria (reference standard)	Targeted population	Disease stage	Initial or recurrent metastasis
Chen Baolin (2024)	Retrospective	China	116 patients (125 lymph nodes)	39:77	17-77/42 ± 12	Pathology	After total thyroidectomy	No reported	Recurrence
Shui-Qing Liu (2023)	Retrospective	China	208 patients (208 lymph nodes)	141:67	43.3 ± 11.9	Pathology	Before thyroid surgery	No reported	Unclear from the article
Santa D'angeli (2023)	Prospective	France	32 patients (58 lymph nodes)	29:3	26.7-69.1/ 47.9	Pathology	Before thyroid surgery	No reported	Unclear from the article
Zhai L (2022)	Prospective	China	443 lymph nodes (preoperative 401, postoperative hemithyroidectomy 42)	nil	43.5	Pathology	Thyroid gland present	No reported	Unclear from the article
			74 patients (74 lymph nodes)				After total thyroidectomy	No reported	Recurrence
Xiaoli Wu (2022)	Prospective	China	58 patients (147 lymph nodes)	59:12	Metastatic, 31-48/39; benign, 38-50/44.5	Pathology	Before thyroid surgery	No reported	Unclear from the article
			13 patients (29 lymph nodes)				After total thyroidectomy	No reported	Recurrence
Jingjing Sun (2022)	Retrospective	China	122 patients (122 lymph nodes)	85:37	NA/49.7	Pathology	After hemithyroidectomy or partial thyroidectomy	No reported	Unclear from the article
			67 patients (67 lymph nodes)	46:21	NA/49.6		After total thyroidectomy	No reported	Recurrence
Xi Jia (2022)	Retrospective	China	228 patients (299 lymph nodes, [136 central lymph nodes; 163 lateral lymph nodes])	165:63	20-81 / 43.8 ± 13.85	Pathology	Before thyroid surgery	No reported	Unclear from the article
Helmi Khadra (2019)	Retrospective	United States	138 patients (unknown number of lymph nodes)	106:32	53.1 ± 14.2	Pathology	After total thyroidectomy	No reported	Recurrence
Zahraa Al-Hilli (2017)	Prospective	United States	480 patients (unknown number of lymph nodes)	298:182	44 ± 16.8	Pathology	After total thyroidectomy	No reported	Recurrence
Jun Ho Lee (2016)	Prospective	Korea	61 patients (225 lymph nodes)	NA	NA	Pathology	Before thyroid surgery	No reported	Unclear from the article
			17 patients (57 lymph nodes)	52:26	19-71/43.0		After total thyroidectomy	No reported	Recurrence
Jia-hong Shi (2015)	Prospective	China	148 patients (148 lymph nodes)	NA	NA	Pathology	Before thyroid surgery	No reported	Unclear from the article
Brittany J. Holmes (2014)	Retrospective	United States	19 patients (21 lymph nodes)	12:7	21-73/46	Pathology	After total thyroidectomy	No reported	Recurrence
Young Joo Suh (2013)	Retrospective	Korea	43 patients (47 lymph nodes)	37:6	23-83/44.9	Pathology	After thyroid surgery	No reported	Recurrence
Jae Hoon Moon (2013)	Retrospective	Korea	72 patients (87 lymph nodes)	388:140	37-67/52	Pathology	Before thyroid surgery	No reported	Unclear from the article
			347 patients (441 lymph nodes)				After total thyroidectomy	No reported	Recurrence
Qing Kay Li (2013)	Prospective	United States	144 patients (208 lymph nodes)	136:72 (lymph nodes)	Benign, 44.9; metastatic, 45.1	Pathology	After thyroid surgery	No reported	Recurrence
Min Ji Jeon (2013)	Retrospective	United States	207 patients (263 lymph nodes)	149:58	12-75/47	Pathology	After total thyroidectomy	No reported	Recurrence
Y.M. Sohn (2012)	Retrospective	Korea	92 patients (95 lymph nodes)	NA	NA	Pathology	Before thyroid surgery	No reported	Unclear from the article
Dae-Weung Kim (2012)	Prospective	Korea	55 patients (73 lymph nodes)	42:13	53.1 ± 15.3	Pathology	Before thyroid surgery	No reported	Unclear from the article
			13 patients (18 lymph nodes)	10:3	41.2 ± 14.2		After total thyroidectomy	No reported	Recurrence
Luca Giovanella (2011)	Prospective	Switzerland	108 patients (126 lymph nodes)	89:19	42.7 ± 18.2	Pathology	After total thyroidectomy	pT1 in 34 cases; pT2 in 43; pT3 in 24; pT4 in 4. Lymph nodes meta-stasis in 45 cases; overall disease stage not reported	Recurrence
André B. Zanella (2010)	Prospective	Brazil	43 patients (43 lymph nodes)	30:13	51.4 ± 14.6	Pathology	Before thyroid surgery	Stage I: 21 patients; stage II: 8; stage III: 12; stage IV: 2	Recurrence
Young Hen Lee (2010)	Retrospective	Korea	40 patients (40 lymph nodes)	32:8	23-68/44	Pathology	After total thyroidectomy	No reported	Recurrence: 21 patients; nonrecurrence: 19
Min Jung Kim (2009)	Prospective	Korea	100 patients (100 lymph nodes)	NA	NA	Pathology	Before thyroid surgery	No reported	Unclear from the article
			68 patients (68 lymph nodes)				After total thyroidectomy	No reported	Recurrence

Abbreviation: NA, not available.

**Table 2.** Quantitative data extracted from included studies. Table presenting FNA-Tg cutoff values and diagnostic 2 × 2 data from included studies. Data include washout method, Tg values, true/false positives and negatives, and calculated positive predictive value and negative predictive value

First author (year)	Fine needle		Serum		Washout fluid		FNA-Tg cutoff value	Diagnostic 2 × 2 table				Positive predict value (%)	Negative predict value (%)
	Thyroglobulin (✓, ✗)	Antithyroglobulin (✓, ✗)	Thyroglobulin (✓, ✗)	Antithyroglobulin (✓, ✗)	Type	Volume	Original value in the primary study (ng/mL, ng/puncture)	True positives	False positives	False negatives	True negatives		
Chen Baolin (2024)	✓	✗	✓	✓	Normal saline	2 mL	4.565 ng/mL	95	0	11	19	100.0	63.3
Shui-Qing Liu (2023)	✓	✗	✓	✗	Normal saline	1 mL	61.6 ng/mL	69	22	10	107	75.8	91.5
Santa D'angeli (2023)	✓	✗	✓	✓	Normal saline	1 mL	60 ng/mL	29	2	9	18	93.5	66.7
Zhai L (2022), thyroid (−)	✓	✗	✓	✓	Normal saline	1 mL	1.2 ng/mL	49	3	2	20	94.2	90.9
Zhai L (2022), thyroid (+)							19.4 ng/mL	270	11	19	143	96.1	88.3
Xiaoli Wu (2022)	✓	✓	✓	✓	Normal saline	1 mL	227.1 ng/mL	18	4	4	32	81.8	88.9
							6.8 ng/mL	7	0	0	6	100.0	100.0
Jingjing Sun (2022)	✓	✗	✓	✓	Normal saline	1 mL	2.3 ng/mL	76	0	3	43	100.0	93.5
							0.743 ng/mL	41	1	1	24	97.6	96.0
Xi Jia (2022)	✓	✗	✓	✓	Total of 5 µL of sample was added to 195 µL of thyroglobulin-free serum	0.195 mL	119.85 ng/mL (central lymph node)	30	30	12	63	50.0	84.0
							16.8 ng/mL (lateral lymph node)	65	3	4	90	95.6	95.7
Helmi Khadra (2019)	✓	✗	✓	✗	Normal saline	1 mL	1 ng/mL	68	2	3	49	97.1	94.2
Zahraa Al-Hilli (2017)	✓	✗	✗	✗	Normal saline	0.1-0.5 mL	1 ng/mL	96	3	5	1	97.0	16.7
Jun Ho Lee (2016)	✓	✗	✓	✓	Normal saline	1 mL	2.2 ng/mL	98	13	21	93	88.3	81.6
							0.6 ng/mL	25	3	1	28	89.3	96.6
Jia-hong Shi (2015)	✓	✗	✓	✗	NA	NA	10 ng/mL	93	17	1	37	84.5	97.4
Brittany J. Holmes (2014)	✓	✗	✗	✗	Hank balanced salt solution without heparin	1 mL	0.2 ng/mL	10	1	0	10	90.9	100.0
Young Joo Suh (2013)	✓	✗	✓	✓	Normal saline	1 mL	1 ng/mL	34	4	0	9	89.5	100.0
Jae Hoon Moon (2013)	✓	✗	✓	✓	Normal saline	1 mL	2.24 ng/mL	31	2	1	53	93.9	98.1
							1.09 ng/mL	143	10	15	273	93.5	94.8
Qing Kay Li (2013)	✓	✗	✓	✓	Hanks’ balanced salt solution without heparin	1 mL	0.2 ng/mL	35	26	1	112	57.4	99.1
Min Ji Jeon (2013)	✓	✗	✓	✓	Normal saline	1 mL	10 ng/mL	82	24	6	151	77.4	96.2
Y. M. Sohn (2012)	✓	✗	✗	✗	Normal saline	1 mL	5 ng/mL	29	9	13	44	76.3	77.2
Dae-Weung Kim (2012)	✓	✗	✗	✗	Normal saline	0.5 mL	50 ng/mL	38	2	3	30	95.0	90.9
								8	2	0	8	80.0	100.0
Luca Giovanella (2011)	✓	✗	✓	✓	Normal saline	1 mL	1.1 ng/mL	80	0	0	35	100.0	100.0
André B. Zanella (2010)	✓	✗	✗	✗	Normal saline	1 mL	10 ng/mL	5	0	0	38	100.0	100.0
Young Hen Lee (2009)	✓	✗	✗	✗	Normal saline	1 mL	4.1 ng/mL	21	0	0	19	100.0	100.0
Min Jung Kim (2009)	✓	✗	✗	✗	Normal saline	1 mL	10 ng/mL	54	5	7	34	91.5	82.9
							ng/mL	56	1	2	9	98.2	81.8

Abbreviations: FNA-Tg, fine-needle aspiration with thyroglobulin washout; NA, not available; thyroid (−), thyroid gland absent; thyroid (+), thyroid gland present.

### Quality Assessment

**Figure 2.**
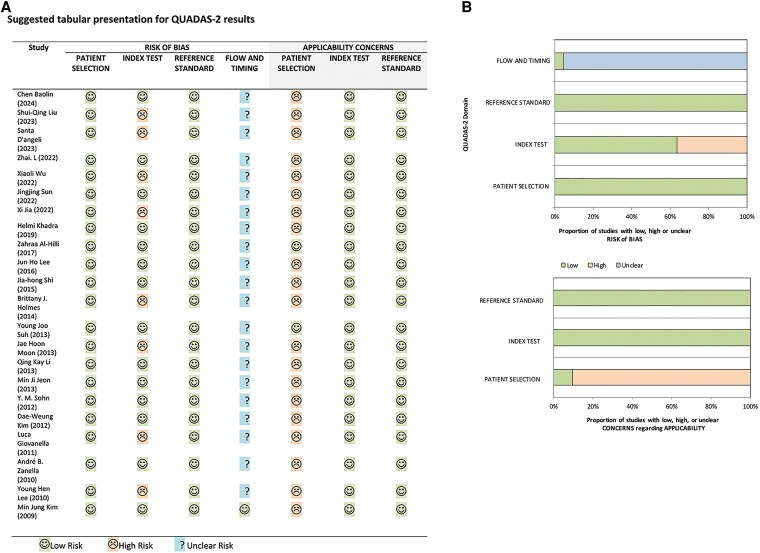
Quality assessment of included studies. Study quality was assessed using Quality Assessment of Diagnostic Accuracy Studies criterion. Quality assessment results in (A) tabular form and (B) graphic form, with each bar representing the percentage of studies considered as having high, low, or unclear risks of bias and poor applicability.

A total of 22 studies published between 2008 and 2024 were included. Methodological quality was assessed using the QUADAS-2 tool. Surgical histopathology served as the reference standard, and FNA-Tg prediction of lymph node status was categorized into true positive, false positive, false negative, or true negative. Eight studies were rated as having a high risk of bias in the index test domain because they set the FNA-Tg cutoff on the basis of their own receiver operating characteristic curves. Studies often had an unclear risk of bias in the flow and timing domain because they did not report the interval between FNA-Tg sampling and surgical confirmation. With regard to applicability concerns in patient selection, only 2 studies were judged as having a low risk of bias because they clearly reported their data on disease severity, comorbidities, and demographic characteristics and on their study setting and prior testing protocols. Overall, most studies had a low risk of bias. A summary of the quality assessment is presented in Fig. 2.

### Diagnostic Performance

**Figure 3.**
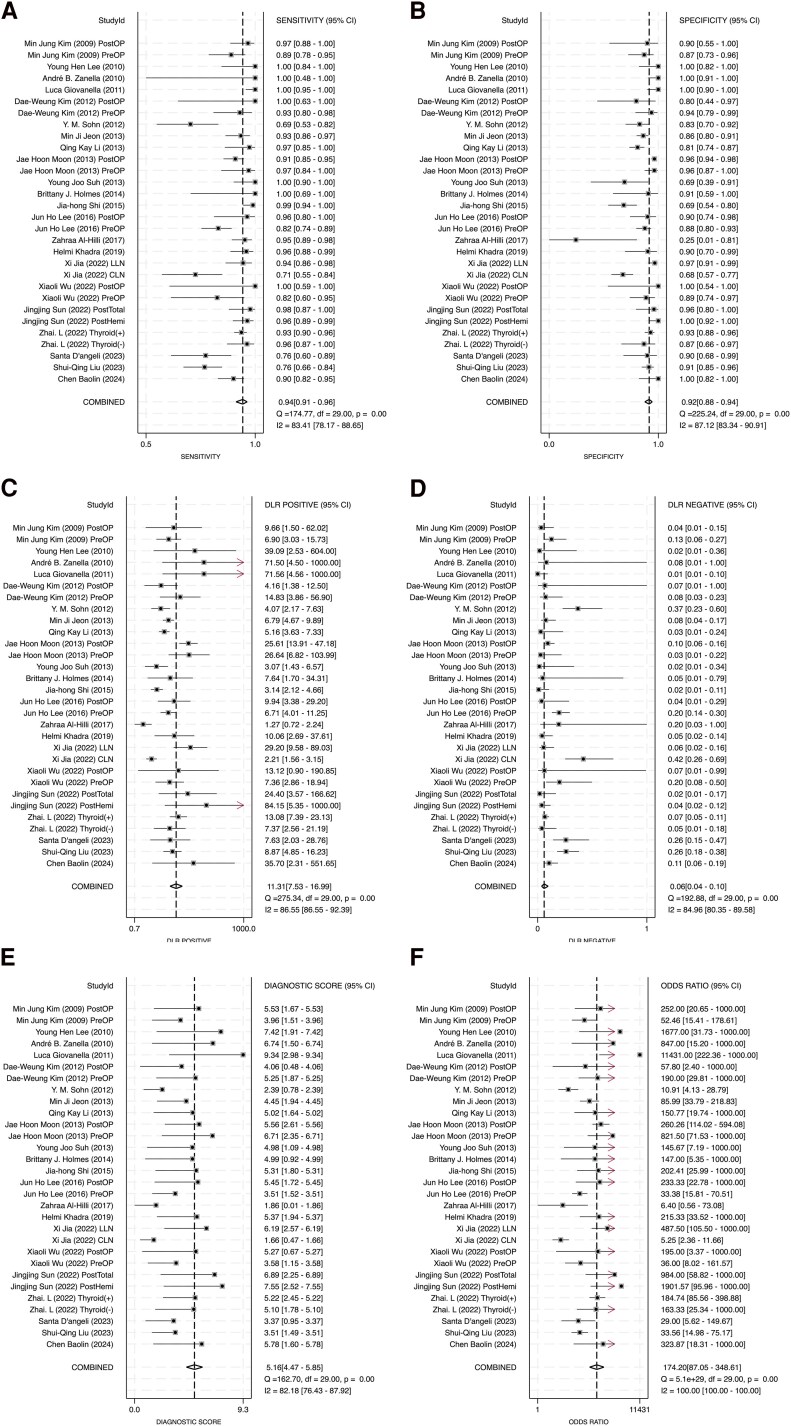
Diagnostic performance of FNA-Tg. (A) Sensitivity, (B) specificity, (C) positive diagnostic likelihood ratio, (D) negative diagnostic likelihood ratio, (E) diagnostic score, (F) diagnostic odds ratio. Abbreviation: FNA-Tg, fine-needle aspiration with thyroglobulin washout.

To assess the diagnostic efficacy of FNA-Tg, we calculated pooled sensitivities, specificities, positive and negative diagnostic likelihood ratios, DORs, PPVs, and NPVs. Across 22 studies, the pooled sensitivity and specificity values were 0.94 (95% CI: 0.91-0.96; Fig. 3A) and 0.92 (95% CI: 0.88-0.94; Fig. 3B), respectively. Substantial heterogeneity was noted (22), with I^2^ values of 83.41% for sensitivity and 87.12% for specificity. The Q-statistics also indicated significant heterogeneity (Q = 174.77, *P* < .001 for sensitivity; Q = 225.24, *P* < .001 for specificity), indicating variability among the included studies. Despite this variability, the high pooled estimates suggested strong diagnostic accuracy. Nevertheless, caution is warranted when generalizing these results due to the underlying heterogeneity.

DLR+ values represent how much the odds of having PTC increase when the test is positive (23). The pooled DLR+ was 11.31 (95% CI: 7.53-16.99; Fig. 3C), exceeding the conventional threshold of 10 and indicating a strong ability to confirm PTC when the FNA-Tg result is positive (24). Nevertheless, significant heterogeneity was present (I^2^ = 86.55%; Q = 275.34, *P* < .001), suggesting variability across studies. The pooled DLR− was 0.06 (95% CI: 0.04-0.10; Fig. 3D), indicating strong rule-out potential for PTC when the test is negative (24). Similar to DLR+, DLR− also demonstrated substantial heterogeneity (I^2^ = 84.96%; Q = 192.88, *P* < .001). Despite this heterogeneity, both metrics supported the robust diagnostic utility of FNA-Tg.

Diagnostic score (DS) and DOR were used to assess the overall diagnostic accuracy of the test. DS, calculated as the natural logarithm of DOR, provides a standardized index for comparing diagnostic accuracy across studies. The pooled DS was 5.16 (95% CI: 4.47-5.85; Fig. 3E), indicating high diagnostic accuracy, although substantial heterogeneity was observed (I^2^ = 82.18%; Q = 162.7, *P* < .001). DOR, representing the odds of a positive test result in patients with disease vs those without (25), was pooled at 174.2 (95% CI: 87.05-348.61; Fig. 3F), suggesting excellent diagnostic power (26). Both DS and DOR confirmed the strong performance of FNA-Tg in confirming malignant cervical lymph nodes in patients with PTC.

PPV and NPV were pooled to further evaluate the clinical utility of FNA-Tg in real-world settings. The pooled PPV was 0.97 [95% CI: 0.96-0.98; Supplemental Fig. S1A (20), (Zenodo)], and the pooled NPV was 0.96 [95% CI: 0.95-0.97; Supplemental Fig. S1B (20), (Zenodo)], indicating excellent rule-in and rule-out performance for detecting PTC lymph node metastases.

**Figure 4.**
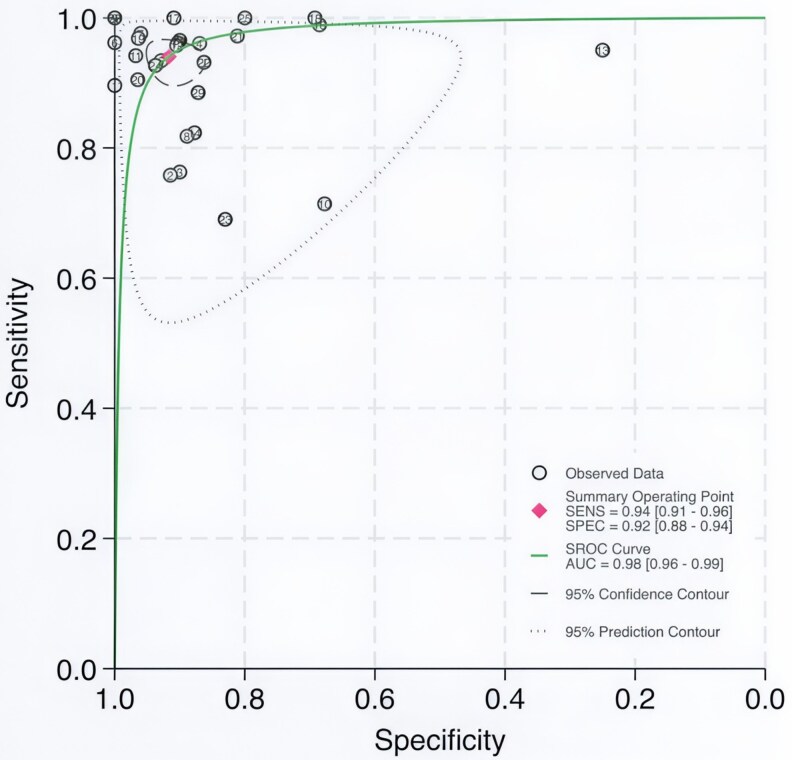
HSROC curve of FNA-Tg in diagnosing suspicious lymph node metastasis in PTC. Abbreviations: FNA-Tg, fine-needle aspiration with thyroglobulin washout; HSROC, hierarchical summary receiver operating characteristic; PTC, papillary thyroid carcinoma.

HSROC curves were used to evaluate the overall diagnostic performance of FNA-Tg. The summary operating point demonstrated a pooled sensitivity of 0.94 (95% CI: 0.91-0.96) and a specificity of 0.92 (95% CI: 0.88-0.94), with an AUC of 0.98 (95% CI: 0.96-0.99), indicating excellent diagnostic accuracy (Fig. 4).

### Threshold Effect

**Figure 5.**
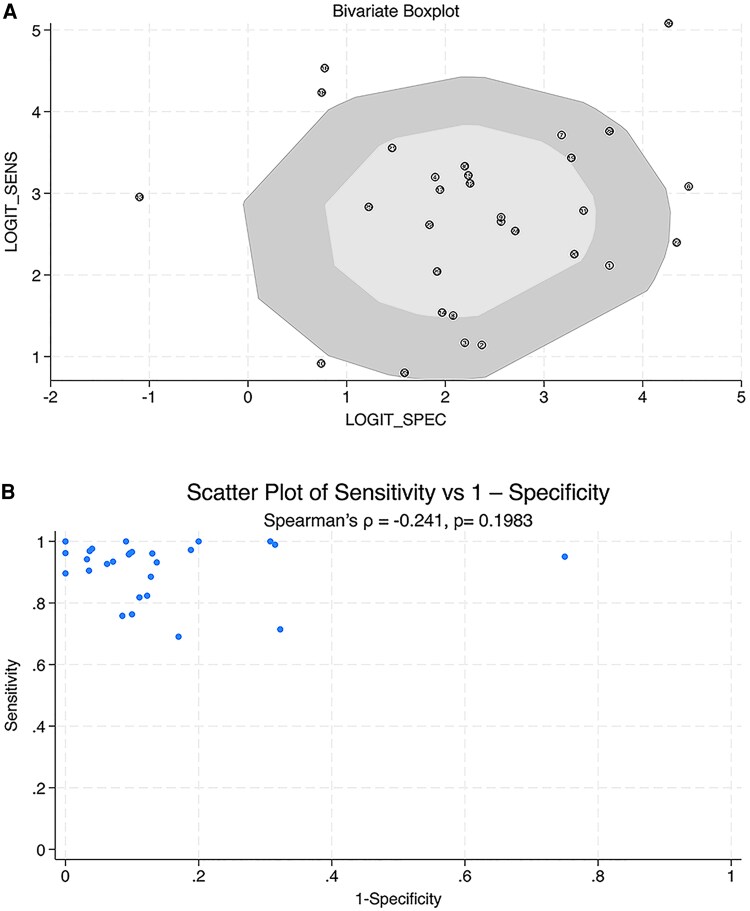
Threshold effect of pooled study. (A) Bivariate boxplot of 22 included studies. (B) Scatter plot of sensitivity vs 1—specificity for evaluating the threshold effect in included studies.

A bivariate boxplot was used to assess the presence of a threshold effect by visualizing the relationship between logit-transformed sensitivity and specificity across studies (Fig. 5A). Most studies clustered within the inner box, indicating consistent diagnostic performance, while a few outliers suggested variability. A mild inverse trend was observed, but Spearman correlation between sensitivity and 1−specificity was −0.241 (*P* = 0.198), indicating no significant threshold effect contributing to heterogeneity (Fig. 5B).

### Subgroup Analysis

**Figure 6.**
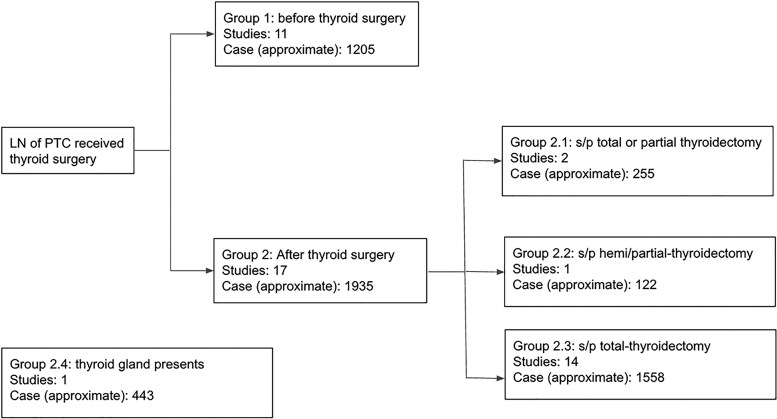
Subgroup analysis. Subgroup classifications were defined as follows: group 1 represented patients in the preoperative state; groups 2.1, 2.2, and 2.3 represented patients in the postoperative state; groups 1, 2.1, 2.2, and 2.4 represented patients thyroid (+); and group 2.3 represented patients thyroid (−). Abbreviations: thyroid (−), thyroid gland absent; thyroid (+), thyroid gland present.

**Figure 7.**
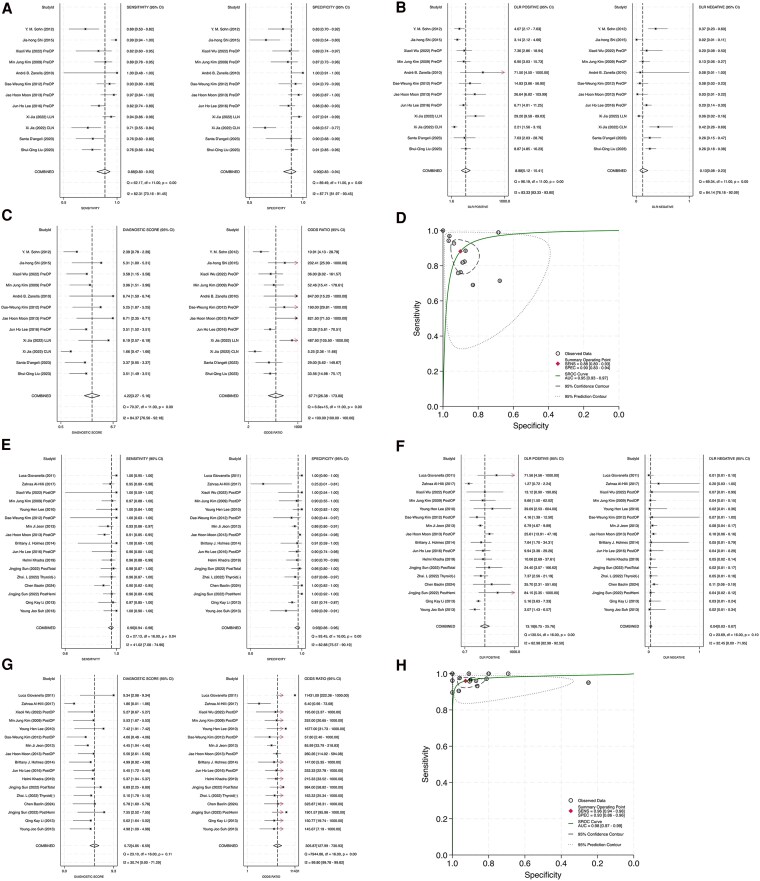
Subgroup analysis by surgical status. (A–D) Diagnostic efficiency in preoperative group. (E–H) Diagnostic efficiency in postoperative group.

Due to variability in patient populations across the studies, we conducted analyses across the following 4 patient subgroups (Fig. 6): preoperative = group 1; postoperative = groups 2.1 + 2.2 + 2.3; thyroid (+) = groups 1 + 2.1 + 2.2 + 2.4; and thyroid (−) = group 2.3. In the preoperative group, the pooled sensitivity and specificity were 0.88 and 0.90, respectively; DLR+ was 8.88; DLR− was 0.13; DS was 4.22; and DOR was 67.71 (Fig. 7A–7C). The HSROC curve showed an AUC of 0.95 (Fig. 7D). Furthermore, the PPV was 0.90, and the NPV was 0.94 [Supplemental Fig. S2 (20), (Zenodo)]. In the postoperative group, the pooled sensitivity and specificity were 0.96 and 0.93, respectively, DLR+ was 13.18, DLR− was 0.04, DS was 5.72, and DOR was 305.87 (Fig. 7E–7G). The AUC of the HSROC was 0.98 (Fig. 7H). The PPV and NPV were 0.98 and 0.97, respectively [Supplemental Fig. S3 (20), (Zenodo)]. Compared with the preoperative group, the postoperative group had higher sensitivity, specificity, DLR+, DS, DOR, PPV, and NPV and lower DLR−. The steeper slope of the HSROC curve in the postoperative group indicated less variability and more consistent diagnostic performance across the studies.

**Figure 8.**
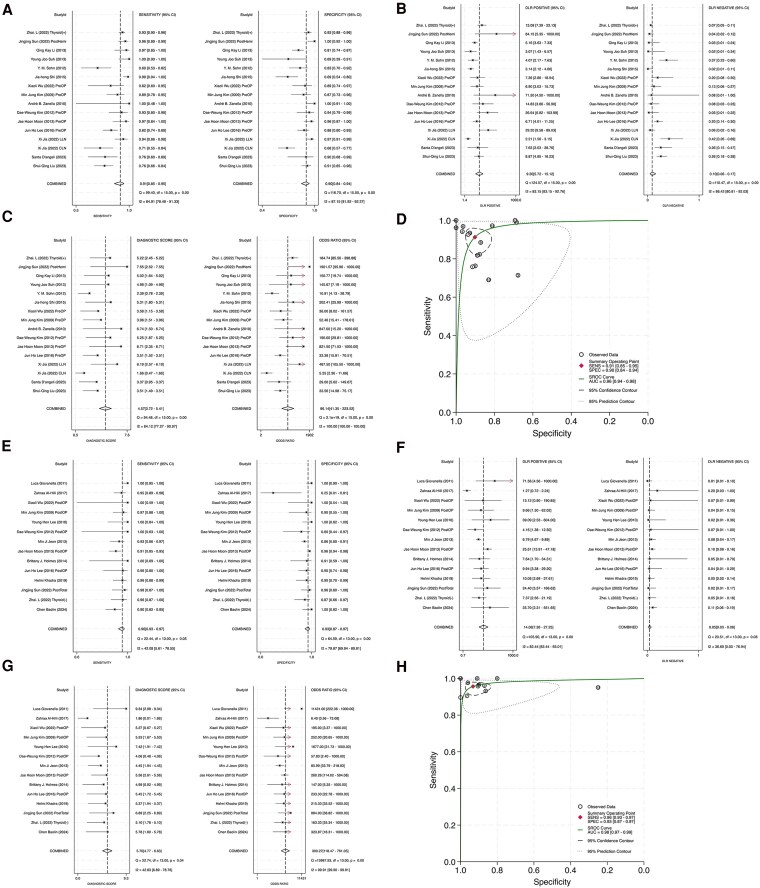
Subgroup analysis in thyroid (+) group and thyroid (−) group. (A–D) Diagnostic efficiency in thyroid (+) group. (E–H) Diagnostic efficiency in thyroid (−) group. Abbreviations: thyroid (−), thyroid gland absent; thyroid (+), thyroid gland present.

Thyroid tissue can interfere with FNA-Tg (27); therefore, the diagnostic performance of FNA-Tg was compared between the thyroid (+) and thyroid (−) groups (Fig. 8). In the thyroid (+) group, the pooled sensitivity and specificity were 0.91 and 0.90, respectively; DLR+ was 9.30; DLR− was 0.10; DS was 4.57; and DOR was 96.14 (Fig. 8A–8C). The HSROC curve showed an AUC of 0.96 (Fig. 8D). Moreover, the PPV was 0.95, and the NPV was 0.96 [Supplemental Fig. S4 (20), (Zenodo)]. By contrast, the thyroid (−) group had higher diagnostic accuracy. The group had pooled sensitivity and specificity values of 0.96 and 0.93, respectively; a DLR+ of 14.06; a DLR− of 0.05; a DS of 5.70; a DOR of 300.27 (Fig. 8E–8G), a PPV of 0.98; and a NPV of 0.95 [Supplemental Fig. S5 (20), (Zenodo)]. The HSROC curve in this group had a higher AUC (0.98) and a steeper slope (Fig. 8H), reflecting greater consistency and diagnostic reliability. Overall, the absence of thyroid tissue was associated with improved diagnostic performance.

### Clinical Utility

**Figure 9.**
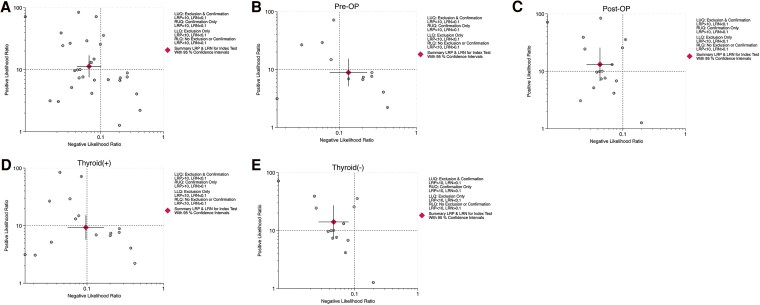
Clinical applicability of FNA-Tg in diagnosing suspicious lymph node metastasis in patients with PTC. Likelihood ratio scattergram showing diagnostic performance of FNA-Tg in exclusion and confirmation. (A) All patients, (B) preoperative group, (C) postoperative group, (D) thyroid (+) group, and (E) thyroid (−) group. Abbreviations: FNA-Tg, fine-needle aspiration with thyroglobulin washout; PTC, papillary thyroid carcinoma; thyroid (−), thyroid gland absent; thyroid (+), thyroid gland present.

A likelihood ratio scattergram was used to evaluate values obtained using different assays (28, 29). The scattergram illustrated the summary estimates of positive and negative likelihood ratios derived from the pooled sensitivity and specificity. In the overall analysis, the summary point was plotted in the left upper quadrant (Fig. 9A), indicating that FNA-Tg was effective for both confirming and excluding lymph node metastases in PTC. In subgroup analyses, the preoperative group had a summary point in the right lower quadrant (Fig. 9B), suggesting limited diagnostic utility. By contrast, the postoperative group's summary point appeared in the left upper quadrant (Fig. 9C), supporting strong diagnostic performance for both confirmation and exclusion. The thyroid (+) group was plotted in the left lower quadrant (Fig. 9D), indicating that FNA-Tg was more useful for exclusion than confirmation. Conversely, the thyroid (−) group was positioned in the left upper quadrant (Fig. 9E), as was the postoperative group, demonstrating high diagnostic accuracy for both confirming and excluding metastatic lymph nodes. These findings suggest that the absence of thyroid tissue enhances the clinical performance of FNA-Tg in identifying nodal metastases or recurrence in PTC.

**Figure 10.**
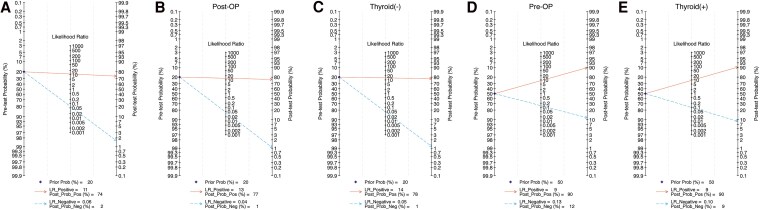
Clinical application of FNA-Tg in Fagan plot. (A) All patients, (B) postoperative group, (C) thyroid (−) group, (D) preoperative group, and (E) thyroid (+) group. Abbreviations: FNA-Tg, fine-needle aspiration with thyroglobulin washout; thyroid (−), thyroid gland absent; thyroid (+), thyroid gland present.

Fagan plot (30) was constructed to evaluate the diagnostic performance of FNA-Tg. In the pooled results, based on a 20% pretest probability, the positive posttest probability was 74% and negative posttest probability was 2% (Fig. 10A). In the postoperative and thyroid (−) subgroups, the positive posttest probabilities were 77% (Fig. 10B) and 78% (Fig. 10C), respectively, and the negative posttest probability was 1% for both. In the preoperative and thyroid (+) subgroups, based on a 50% pretest probability, the positive posttest probabilities were both 90% (Fig. 10D and 10E) and negative posttest probabilities were 12% and 9%, respectively. Overall, FNA-Tg demonstrated remarkable clinical applicability in the diagnostic evaluation of metastatic or recurrent lymph nodes in PTC.

### Associations of FNA-Tg Measurement With s-Tg and s-Tg-Ab

**Figure 11.**
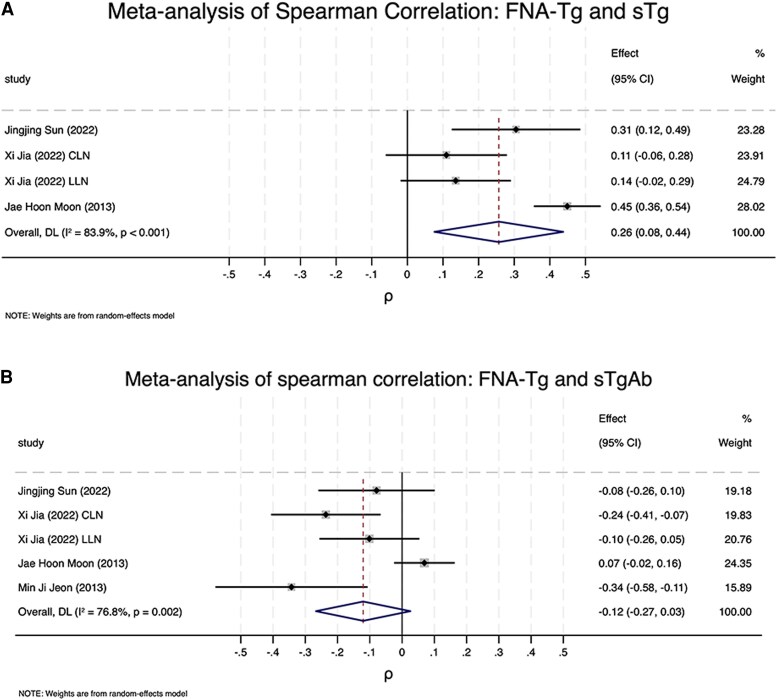
Associations of FNA-Tg measurements with s-Tg and s-Tg-Ab concentrations. (A) Meta-analysis of Spearman's correlation for relationship between FNA-Tg and s-Tg. (B) Meta-analysis of Spearman's correlation for relationship between FNA-Tg and s-Tg-Ab. Abbreviations: FNA-Tg, fine-needle aspiration with thyroglobulin washout; s-Tg, serum thyroglobulin; s-Tg-Ab, serum thyroglobulin antibody.

Among the 22 included studies, 4 provided data on FNA-Tg measurements and s-Tg, while 5 studies provided data on FNA-Tg and s-Tg-Ab. A meta-analysis of Spearman's correlation findings revealed a significant but weak positive correlation between FNA-Tg measurements and s-Tg concentrations (ρ = .26; 95% CI: 0.08-0.44; *P* < .001; Fig. 11A). By contrast, a weak inverse correlation was observed between FNA-Tg measurements and s-Tg-Ab concentrations (ρ = −.12; 95% CI: −0.27 to 0.03; *P* = .002); however, the CI included 0, indicating that the correlation was not significant (Fig. 11B). These findings suggest that FNA-Tg measurements are modestly correlated with s-Tg concentrations but not with s-Tg-Ab concentrations.

### Sensitivity Analysis

To assess the robustness of diagnostic estimates, sensitivity analyses were performed by excluding studies with a high risk of bias across the overall cohort and all predefined subgroups. Each study was sequentially removed to evaluate its individual influence on the pooled results, regardless of whether the effect was positive or negative [Supplemental Figs. S6–S10 (20), (Zenodo)]. The most influential study in each subgroup was then excluded, and the pooled diagnostic estimates were recalculated to determine whether substantial changes occurred [Supplemental Figs. S11–S15 (20), (Zenodo)]. Comparisons were conducted between results before and after sensitivity analysis across all 5 subgroups. The results remained consistent, with minimal changes in diagnostic accuracy and improved heterogeneity in most parameters. In the pooled group, sensitivity and specificity remained at 0.94 and 0.93, respectively. DLR+, DLR−, and predictive values (PPV: 0.97, NPV: 0.96) were unchanged, while DS and DOR increased slightly with improved consistency. Across all subgroups—including the preoperative group, postoperative group, thyroid (+) group, and thyroid (−) group—diagnostic estimates were stable. The preoperative group showed a modest decrease in sensitivity (0.88 → 0.84), whereas the other subgroups demonstrated nearly identical results. Heterogeneity was notably reduced in metrics such as DLR+ and DS, further supporting the reliability of the pooled findings. These results confirm that the diagnostic performance of FNA-Tg is statistically robust and generalizable across diverse clinical contexts.

### Publication Bias

**Figure 12.**
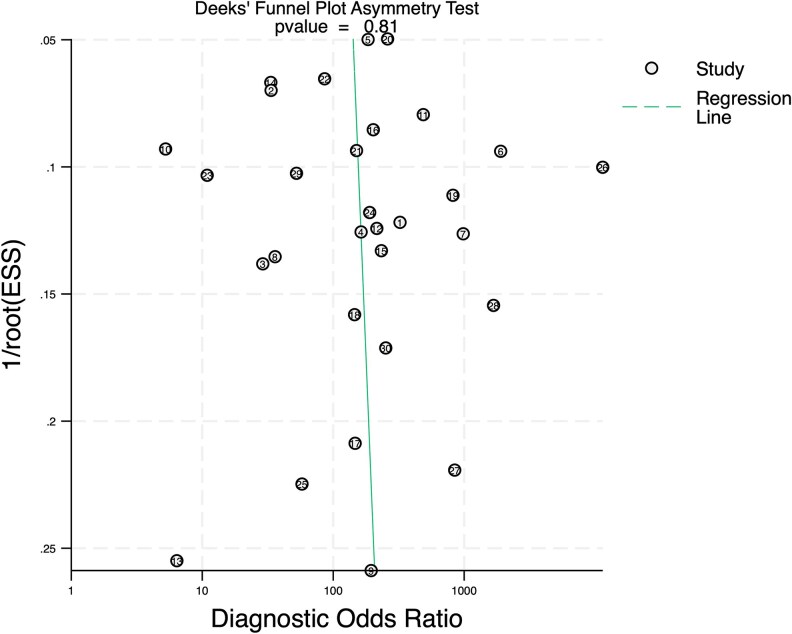
Deeks’ funnel plot asymmetry test in 22 studies evaluating use of FNA-Tg for diagnosing suspicious metastatic lymph nodes in PTC. Abbreviations: FNA-Tg, fine-needle aspiration with thyroglobulin washout; PTC, papillary thyroid carcinoma.

A Deeks’ funnel plot was used to evaluate publication bias (31, 32). Significant publication bias in the pooled results was not observed (*P* = .81; Fig. 12). Therefore, the pooled estimates of sensitivity, specificity, DOR, and other diagnostic metrics were unlikely to be influenced by selective reporting and were considered representative of the true diagnostic performance of the test.

## Discussion

This meta-analysis of 22 studies confirmed the excellent diagnostic performance of FNA-Tg for detecting metastatic lymph nodes in patients with PTC, with pooled sensitivity and specificity values of 0.94 and 0.92, respectively, and an AUC of 0.98. These metrics surpassed those obtained in other studies, which have reported broader ranges of sensitivity (0.69-1.00), specificity (0.50-1.00), and AUC (0.76-1.00) (14). Nevertheless, substantial heterogeneity in sensitivity was observed (I^2^ = 83.41%), not due to a threshold effect, which was identified using bivariate boxplot and Spearman analysis (33). This heterogeneity necessitated subgroup analyses. Notably, FNA-Tg performed best when used with patients without thyroid glands [postoperative and thyroid (−) groups], achieving sensitivity and specificity values of 0.96 and 0.93, respectively, and an AUC of 0.98. This finding highlights the enhanced diagnostic value of FNA-Tg in patients who have undergone total thyroidectomy, hemithyroidectomy, or partial thyroidectomy. A weak positive correlation between FNA-Tg measurements and s-Tg concentrations was observed. A significant correlation between FNA-Tg measurements and s-Tg-Ab concentrations was not observed, suggesting minimal interference from s-Tg-Ab and supporting FNA-Tg as a reliable marker for PTC lymph node metastasis.

Treatment guidelines of the American Thyroid Association and European Thyroid Association strongly recommend the use of FNA-Tg to assess the presence of lymph node metastasis in patients without thyroid glands (10, 34). Several studies have noted that FNA-Tg was the primary method of identifying lymph node metastasis in patients with PTC who had not yet undergone thyroidectomy (35-37). By contrast, previous studies conducting postoperative surveillance have typically employed s-Tg measurements to detect persistent or recurrent PTC (38-42). Postoperative s-Tg measurements are affected by numerous factors, including the extent of remaining thyroid cancer or normal thyroid tissue, serum thyroid-stimulating hormone concentrations, the analytic sensitivity of the s-Tg detection method, selected s-Tg threshold values, individual patient risk for radioactive iodine-avid local or distant metastasis, interval time from total thyroidectomy, and the diagnostic sensitivity of posttherapy imaging modalities (10). Given the limitations of s-Tg in reliably detecting recurrent disease, the diagnostic utility of FNA-Tg for recurrent PTC has garnered considerable interest. Other studies have indicated that FNA-Tg represents a viable alternative to fine-needle aspiration cytology due to its affordability, simplicity, and other factors. Additionally, FNA-Tg maintains its reliability even in very small lesions, being unaffected by the presence of thyroglobulin antibodies and demonstrating high diagnostic accuracy (37, 43, 44). Despite these factors, 1 study has argued against relying solely on positive FNA-Tg results as an indication for surgical resection, emphasizing that positive cytological findings should remain the primary indication for lymph node excision (37). Furthermore, FNA-Tg is prone to yielding false-negative results in cases of poorly differentiated metastases and false-positive results in cases of blood contamination (36, 45, 46). Consequently, FNA-Tg remains primarily an adjunctive diagnostic tool, while histopathological confirmation through surgical pathology continues to be the gold standard. FNA-Tg can be performed before obtaining tissue samples for definitive diagnosis. FNA-Tg is minimally invasive and provides valuable clues regarding the likelihood of lymph node metastasis. We attempted to further enhance the clinical utility of FNA-Tg. We investigated whether its diagnostic performance depended on the clinical context. Subgroup analyses were conducted to evaluate the diagnostic accuracy of FNA-Tg specifically in preoperative and postoperative settings. Additionally, analyses were conducted to explore how thyroid gland status affects diagnostic efficacy.

The preoperative group in this study had a sensitivity of 0.88, specificity of 0.90, and AUC of 0.95, and the thyroid (+) group had a sensitivity of 0.91, specificity of 0.90, and AUC of 0.96. Notably, the diagnostic performance of FNA-Tg was substantially higher in the postoperative and thyroid (−) groups. These groups had a sensitivity of 0.96, specificity of 0.93, and AUC of 0.98. Other studies have noted that postoperative FNA-Tg measurements, which avoid interference from s-Tg, yield higher sensitivity values, and are more accurate relative to preoperative evaluations (47-50). Because s-Tg predominantly originates from thyroid tissue (47, 51), the effect of s-Tg on FNA-Tg measurements is likely to be smaller in patients who have undergone thyroidectomy. FNA-Tg cutoff values varied considerably between groups, ranging from 2 to 227 ng/mL in the preoperative and thyroid (+) groups and from 0.2 to 10 ng/mL in the postoperative and thyroid (−) groups (Table 2). This difference underscores not only the variability in the setting of the cutoff value across studies but also the tendency for cutoff values to be higher when thyroid tissue is present. A standardized FNA-Tg cutoff value has not been established. One study recommended using a preoperative value of 32.04 ng/mL and a postoperative value of 0.9 ng/mL to identify neck lymph node metastasis (52). Another study proposed an FNA-Tg cutoff value of 2.24 ng/mL in patients who have not yet undergone thyroidectomy and 1.09 ng/mL in those who have undergone thyroidectomy (53), further supporting the results in this study. Numerous studies have demonstrated a positive correlation between FNA-Tg measurements and s-Tg concentrations, suggesting that elevated s-Tg concentrations likely contribute to higher FNA-Tg cutoff values in preoperative and thyroid (+) groups (48, 53, 54).

s-Tg is exclusively synthesized by thyroid follicular cells, making s-Tg an optimal tumor marker for monitoring recurrent or persistent PTC in patients who have undergone thyroidectomy. Excessive s-Tg concentrations might influence the accuracy of FNA-Tg measurements (55, 56). A positive association between s-Tg concentration and measured FNA-Tg value was observed in patients who had not yet undergone thyroidectomy; no such correlation was observed in patients who had undergone thyroidectomy (55). Conversely, several studies observed no significant association between FNA-Tg measurements and s-Tg concentration (48, 57). The contamination of fine-needle aspiration washout fluid with s-Tg is rare, ranging between 0.003% and 0.012% (58). Whether s-Tg affects FNA-Tg remains unclear. The present study observed a weak positive correlation between FNA-Tg measurements and s-Tg concentrations, indicating that the presence of s-Tg may need to be considered when taking FNA-Tg measurements.

Another factor that may influence FNA-Tg cutoff value is s-Tg-Ab. s-Tg-Ab may influence FNA-Tg, with lower FNA-Tg measurements observed in s-Tg-Ab-positive patients than in s-Tg-Ab-negative patients (59). Jia (2022) and Jeon (2013) obtained ρ values of −.24 and −.34, respectively. A weak negative correlation between FNA-Tg measurements and s-Tg-Ab concentration may exist. The results of the present meta-analysis are consistent with those of a study that observed no significant correlation between FNA-Tg measurements and s-Tg-Ab concentration (55). According to the literature, the presence of Tg-Ab in peripheral blood does not interfere with the measurement of thyroglobulin levels in fine-needle aspiration washout fluid. This is likely attributable to the fact that intracellular thyroglobulin within lymph node is not exposed to circulating Tg-Ab (60). Additionally, the binding of s-Tg to s-Tg-Ab prevents s-Tg-Ab from interfering with FNA-Tg (36). The present study demonstrated that a significant correlation does not exist between FNA-Tg measurements and s-Tg-Ab concentration. FNA-Tg may serve as a valuable follow-up tool for patients with PTC who have undergone thyroidectomy.

Likelihood ratios provide valuable insights for diagnostic decision-making. Positive likelihood ratios greater than 10 are considered strong evidence of disease, and negative likelihood ratios lower than 0.1 strongly support ruling out disease (24). The present study's pooled results demonstrated a positive likelihood ratio of 11 and a negative likelihood ratio of 0.06, both meeting the criteria for strong diagnostic evidence. Specifically, in the postoperative and thyroid (−) groups, the positive likelihood ratios were 13 and 14, respectively, and the negative likelihood ratios were 0.04 and 0.05, respectively, indicating superior diagnostic performance compared with the overall pooled results. Given that the lymph node metastasis rate in PTC is 30% to 80% (6) at initial diagnosis and that the postoperative recurrence rate is approximately 20% (7-9), the posttest probability based on Fagan's nomogram was 90% for the preoperative and thyroid (+) groups and 77% and 78% for the postoperative and thyroid (−) groups, respectively. If the test was positive, the probability of metastatic lymph nodes was approximately 90% in the preoperative and thyroid (+) groups and approximately 77% and 78% in the postoperative and thyroid (−) groups, respectively. The relatively low posttest probabilities were likely due to various factors that affect FNA-Tg. On the basis of the present findings, FNA-Tg appears to be a reliable tool for diagnosing and excluding lymph node metastasis in patients with PTC and is only minimally influenced by s-Tg-Ab.

Spearman correlation analysis indicated that the observed heterogeneity was not attributable to a threshold effect. Instead, it is likely driven by study-level confounding factors, including differences in washout fluid types, FNA-Tg measurement protocols, thyroid gland status, s-Tg concentrations, the presence of s-Tg-Ab, and lymph node characteristics (14). Prior evidence suggested that s-Tg (51, 54-56), s-Tg-Ab (58, 59, 61), and remnant thyroid tissue (48-50) may interfere with FNA-Tg quantification and diagnostic performance, thus serving as plausible contributors to interstudy variability. To address this, subgroup analyses were conducted based on surgical status and thyroid tissue presence [ie, preoperative group vs postoperative group; thyroid (+) vs thyroid (−)]. The diagnostic performance of FNA-Tg was consistently higher in the postoperative and thyroid (−) groups, with improvements observed across multiple parameters including sensitivity, specificity, DLR+, DS, DOR, PPV, NPV, and AUC of the HSROC curve. These findings suggested that eliminating confounding from residual thyroid tissue and circulating biomarkers may enhance diagnostic clarity and reduce heterogeneity. To further evaluate the robustness of these findings, sensitivity analyses were performed using a leave-one-out approach within each subgroup. The most influential studies were identified and excluded, and the resulting pooled estimates remained consistent. Notably, in the postoperative and thyroid (−) subgroups, heterogeneity (I^2^) further decreased following study exclusion. These results reinforced the stability and internal validity of the subgroup-specific findings and highlighted the clinical relevance of stratifying by surgical and thyroid status in optimizing FNA-Tg–guided diagnosis.

This study had several limitations. First, the retrospective literature search strategy may have introduced selection bias. Second, variability in the time interval between FNA-Tg (index test) and surgical pathology, which was the reference standard, may have affected diagnostic accuracy. Additionally, due to methodological constraints inherent to diagnostic meta-analyses, determining an accurate FNA-Tg cutoff value was challenging. Alternative strategies may be necessary to establish clinically applicable thresholds for identifying metastatic lymph nodes in both newly diagnosed and recurrent PTC. Additionally, this meta-analysis was based on study-level aggregate data, which precluded the generation of calibration curves or the calculation of Brier scores. These metrics require individual patient-level data to evaluate the agreement between predicted probabilities and observed outcomes. Future studies incorporating patient-specific FNA-Tg values are warranted to assess calibration and enhance risk stratification. Moreover, this study did not assess the potential effect of therapeutic interventions such as radioactive iodine therapy, targeted therapies, and chemotherapy on the diagnostic accuracy of FNA-Tg in postoperative and thyroid-absent patients with PTC. These treatments may influence FNA-Tg measurements and alter the diagnostic performance of FNA-Tg. Future studies are needed to investigate these variables and further optimize the clinical utility of FNA-Tg in the follow-up and management of recurrent PTC.

## Conclusions

This meta-analysis confirmed the high diagnostic accuracy of FNA-Tg for detecting suspicious cervical lymph node metastases in patients with PTC, including both newly diagnosed and recurrent cases. The pooled sensitivity and specificity values were 0.94 and 0.92, respectively, with an AUC of 0.98. Subgroup analyses showed enhanced performance in patients who had undergone thyroidectomy or without residual thyroid tissue [postoperative and thyroid (−) groups], achieving a sensitivity of 0.96, specificity of 0.93, and AUC of 0.98. This result suggests that the absence of a thyroid gland may improve FNA-Tg accuracy. Likelihood ratio scattergrams demonstrated the tool's robust utility in confirming and excluding metastases in these subgroups; moreover, the Fagan plot supported favorable posttest probabilities. A weak positive correlation between FNA-Tg measurements and s-Tg concentration was observed; no significant association with s-Tg-Ab concentration was observed, indicating minimal interference. No significant publication bias was detected. Overall, FNA-Tg represents a reliable and clinically valuable adjunctive tool for evaluating cervical lymph node metastasis in PTC, especially in a postoperative setting and among patients who have undergone total thyroidectomy.

## Data Availability

Some or all datasets generated during and/or analyzed during the current study are not publicly available but are available from the corresponding author on reasonable request.

## Abbreviations

AUC: area under the curve
rCI: confidence interval
rDLR−: negative diagnostic likelihood ratio
rDLR+: positive diagnostic likelihood ratio
rDOR: diagnostic odds ratio
rDS: diagnostic score
rFNA-Tg: fine-needle aspiration with thyroglobin washout
rHSROC: hierarchical summary receiver operating characteristic
rNPV: negative predictive value
rPPV: positive predictive value
rPTC: papillary thyroid cancer
rQUADAS-2: Quality Assessment of Diagnostic Accuracy Studies-2
rs-Tg: serum thyroglobulin
rs-Tg-Ab: serum thyroglobulin antibodies
rthyroid (−): thyroid gland absent
rthyroid (+): thyroid gland present
